# Effectiveness of an Educational Intervention on Awareness of Breast and Cervical Cancers and Human Papillomavirus Vaccine Among Healthcare Professionals in Primary Health Centers of Davanagere District, Karnataka, India

**DOI:** 10.7759/cureus.105152

**Published:** 2026-03-13

**Authors:** Shashikala N V, Muganagowda Patil, Shubha Davalagi, Basavaraj A C, NVSRR Phani Krishna

**Affiliations:** 1 Pediatrics, Jagadguru Jayadeva Murugarajendra Medical College, Davanagere, IND; 2 Public Health, Jagadguru Jayadeva Murugarajendra Medical College, Davanagere, IND; 3 Preventive Medicine, Jagadguru Jayadeva Murugarajendra Medical College, Davanagere, IND

**Keywords:** awareness, breast cancer, cervical cancer, educational intervention, hpv vaccine, primary health center, screening

## Abstract

Breast and cervical cancers are among the most prevalent malignancies affecting women in India, and despite being largely preventable and treatable when detected early, inadequate public awareness and poor screening practices continue to contribute to high morbidity and mortality. Healthcare professionals working at the primary health center (PHC) level play a crucial role in community education, early detection, and timely referral. This quasi-experimental pre-test and post-test study was conducted among 206 healthcare professionals from 48 PHCs in Davanagere district, Karnataka, India, to evaluate the effectiveness of a structured educational intervention in improving awareness of breast and cervical cancer. Knowledge was assessed using a validated 18-item Kannada questionnaire covering symptoms, risk factors, screening guidelines, breast self-examination, human papillomavirus (HPV) vaccination, and Papanicolaou (Pap) smear practices. Participants underwent a 45-60-minute structured educational session, and pre-test and post-test scores were compared using a paired t-test. The mean pre-test score of 13.33±2.64 improved significantly to 17.43±0.69 following the intervention (p<0.0001). Nursing officers constituted the largest proportion of participants (27%), followed by primary healthcare officers (PHCOs) (22%) and doctors (20%), and a significant improvement in awareness was observed across all professional categories. The findings demonstrate that structured educational interventions are effective in enhancing awareness of breast cancer, cervical cancer, and HPV vaccination among PHC-level healthcare workers, highlighting the importance of regular training programs to strengthen early cancer detection and timely referral to higher healthcare centers.

## Introduction

Breast and cervical cancers represent major public health challenges in India and are among the leading causes of cancer-related morbidity and mortality in women. Current national and global estimates indicate that breast cancer is the most frequently diagnosed malignancy among Indian women, while cervical cancer ranks second, together accounting for a substantial proportion of the overall female cancer burden [1-4]. Despite advances in diagnostic techniques and therapeutic options, survival outcomes in India remain lower than those observed in high-income countries. This disparity is largely attributable to late-stage presentation, a relatively younger age at disease onset, and the absence of effective population-based screening programs [5-7].

The impact of these cancers is further exacerbated by low levels of awareness, sociocultural barriers, stigma, and limited access to preventive healthcare services, particularly in rural and semi-urban regions [5-9]. Although cost-effective preventive strategies such as breast self-examination, Papanicolaou (Pap) smear screening, and human papillomavirus (HPV) vaccination have demonstrated efficacy in reducing disease incidence and mortality, their adoption in India remains suboptimal [7,10-12]. Several studies have reported significant gaps in knowledge, attitudes, and screening practices related to breast and cervical cancer as well as HPV vaccination, not only among women but also among healthcare providers, posing a critical barrier to effective cancer control efforts [8,11,13].

Primary health centers (PHCs) form the backbone of India's public healthcare delivery system and play a pivotal role in preventive care, early diagnosis, health education, and community outreach. Healthcare professionals working at PHCs, including medical officers, nursing officers, community health officers (CHOs), pharmacists, and laboratory technicians, often serve as the first point of contact for women accessing healthcare services. Their level of knowledge, awareness, and confidence significantly influences opportunistic screening practices, patient counselling, vaccination advocacy, and timely referral to higher centers [9,13,14]. Inadequate training and insufficient awareness among frontline healthcare workers have been consistently identified as major impediments to the effective implementation of national cancer screening programs [6,12].

National and international agencies, including the World Health Organization (WHO) and the Government of India through the National Program for Prevention and Control of Cancer, Diabetes, Cardiovascular Diseases, and Stroke, emphasize capacity building of primary-level healthcare professionals as a cornerstone of cancer prevention and early detection strategies [10,12]. Educational interventions aimed at healthcare workers have consistently demonstrated significant improvements in knowledge, attitudes, and screening practices related to breast and cervical cancer, as well as HPV vaccination, across both Indian and global settings [12-14].

The district of Davanagere in Karnataka, India, characterized by a mix of rural, semi-urban, and urban PHCs and a diverse healthcare workforce, provides an appropriate setting to assess existing knowledge gaps and evaluate the impact of targeted educational interventions. Assessing the effectiveness of structured training programs among PHC professionals is essential for informing future capacity-building initiatives and strengthening cancer prevention and early detection efforts at the primary healthcare level.

Against this background, the present study was undertaken to evaluate the effectiveness of a structured educational intervention in improving awareness of breast cancer, cervical cancer, and HPV vaccination among healthcare professionals working in PHCs of Davanagere district. By assessing knowledge levels before and after the intervention, this study aims to provide evidence supporting targeted educational programs as a sustainable strategy for strengthening early detection and prevention of women's cancers within the primary healthcare system.

## Materials and methods

Study design and setting

A quasi-experimental pre-test and post-test study design was employed to assess the effectiveness of a structured educational intervention. The study was conducted across 48 PHCs in Davanagere district, Karnataka, India, encompassing rural, semi-urban, and urban healthcare settings. Approval was obtained from the Institutional Ethics Committee of Jagadguru Jayadeva Murugarajendra Medical College (approval number: JJMMC/IEC-49-2025; date: August 2, 2025).

Study population and sample size

The study population comprised healthcare professionals working in PHCs, including medical officers, nursing officers, primary healthcare officers (PHCOs), pharmacists, laboratory technicians, CHOs, and health inspecting officers (HIOs). A total of 206 healthcare professionals participated in the study and completed both the pre-intervention and post-intervention assessments.

Eligibility criteria

Healthcare professionals working at PHCs who were willing to participate and who completed both the pre-test and post-test assessments were included in the study. Participants were excluded if they submitted incomplete questionnaires, were absent during the training session, or were unwilling to participate.

Study tool

Data were collected using a structured questionnaire developed by the authors specifically for this study to assess awareness and knowledge regarding breast cancer, cervical cancer, and HPV vaccination among healthcare professionals. The questionnaire was formulated based on a review of relevant literature and national guidelines.

The original draft was prepared in English and subsequently translated into Kannada, the local language, to ensure better comprehension among participants. The translated version underwent back-translation into English by an independent bilingual expert to ensure conceptual equivalence. The questionnaire was reviewed by subject experts for content validity and by language experts to ensure clarity, appropriateness, and linguistic accuracy. Necessary modifications were made based on expert feedback prior to final administration.

**Figure 1:**
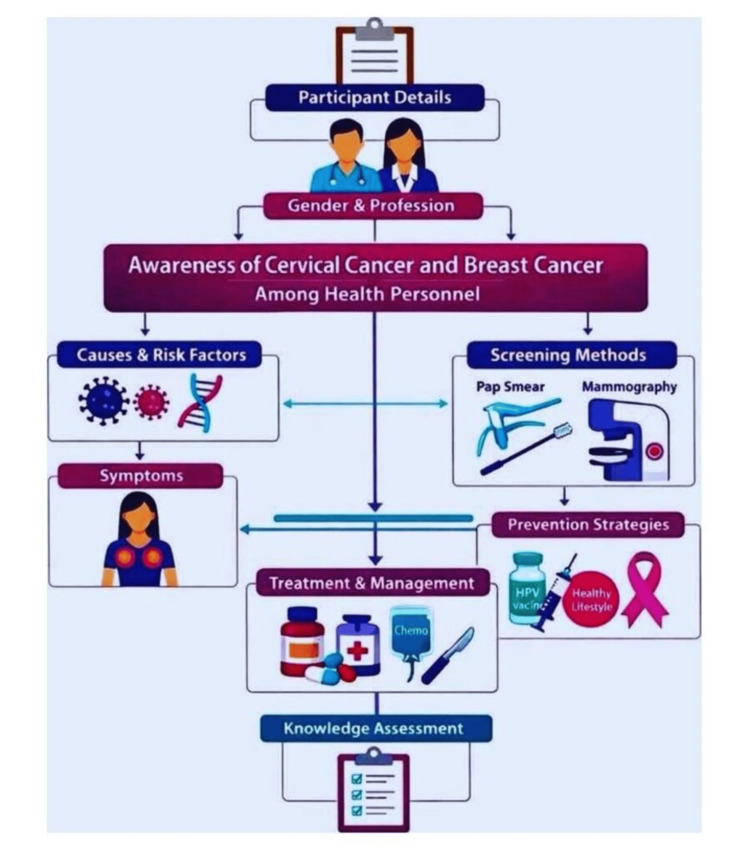
Flowchart depicting the structure of the questionnaire assessing awareness of cervical and breast cancer among health personnel Image created by the authors using Canva

The final questionnaire consisted of 18 items, including multiple-choice and true/false questions, covering domains such as symptoms, risk factors, prevention strategies, screening practices, and vaccination guidelines. Each correct response was awarded 1 point, yielding a maximum possible score of 18. Higher scores indicated better awareness and knowledge levels (Figure 1).

Validation and reliability of the tool

The questionnaire was developed by the authors and subsequently subjected to content validation by subject experts from the Departments of Community Medicine, Obstetrics and Gynecology, and Oncology. The experts evaluated the tool for relevance, clarity, comprehensiveness, and appropriateness of the items in relation to the study objectives. Necessary modifications were incorporated based on their feedback before the finalization of the instrument.

Reliability analysis was performed to assess internal consistency using Cronbach's alpha of 0.83. The obtained Cronbach's alpha value demonstrated acceptable internal consistency, indicating that the tool was reliable and suitable for assessing knowledge in the study population.

Educational intervention

The educational intervention was developed by the authors based on national guidelines, standard screening recommendations, and relevant literature on breast cancer, cervical cancer, and HPV vaccination. The content and structure of the intervention were reviewed by subject experts from the Departments of Community Medicine, Obstetrics and Gynecology, and Oncology to ensure scientific accuracy, relevance, and appropriateness for primary healthcare professionals. Necessary modifications were incorporated based on expert feedback prior to implementation.

The final intervention consisted of a 45-60-minute structured educational program delivered to participants at their respective PHCs. The program included a PowerPoint presentation, a video demonstration of breast self-examination, a detailed explanation of Pap smear screening procedures, HPV vaccination recommendations, and the identification of warning symptoms of breast and cervical cancers. The session concluded with an interactive question-and-answer segment to clarify doubts and reinforce key concepts.

Data analysis

Data were entered and analyzed using IBM SPSS Statistics for Windows, Version 26.0 (IBM Corp., Armonk, New York, United States). Descriptive statistics were used to summarize participant characteristics and knowledge scores. Continuous variables were expressed as mean±standard deviation, while categorical variables were presented as frequencies and percentages. Before applying parametric tests, the distribution of total knowledge scores was assessed for normality using the Shapiro-Wilk test and visual inspection of histograms and Q-Q plots. Since the knowledge scores were derived from summated scale items and demonstrated approximate normal distribution, they were treated as continuous variables. The paired t-test was applied to compare pre-test and post-test knowledge scores to evaluate the effectiveness of the educational intervention. A p-value of <0.05 was considered statistically significant.

## Results

**Figure 2:**
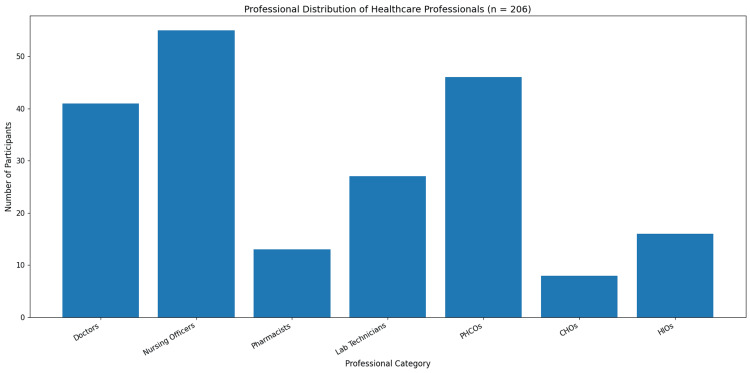
Bar chart showing the professional distribution of healthcare professionals participating in the study The bar chart illustrates the number of participants across different professional categories. Nursing officers constituted the largest group, followed by PHCOs and doctors. PHCOs: primary healthcare officers; CHOs: community health officers; HIOs: health inspecting officers

A total of 206 healthcare professionals participated in the study. As shown in Table 1 and Figure 2, nursing officers constituted the largest group of participants (n=55; 27%), followed by PHCOs (n=46; 22%) and doctors (n=41; 20%). Laboratory technicians accounted for 27 participants (13%), while HIOs and pharmacists comprised 16 (8%) and 13 (6%) participants, respectively. Community health officers represented the smallest group (n=8; 4%).

**Table 1:** Professional distribution of healthcare professionals participating in the study The table shows the distribution of healthcare professionals who participated in the study (n=206), presenting the number and percentage of participants across different professional categories. PHCOs: primary healthcare officers; CHOs: community health officers; HIOs: health inspecting officers

	Number (n=206)	Percentage
Doctors	41	19.9%
Nursing officers	55	26.7%
Pharmacists	13	6.3%
Lab technicians	27	13.1%
PHCOs	46	22.3%
CHOs	8	3.9%
HIOs	16	7.8%
Total	206	100%

**Figure 3:**
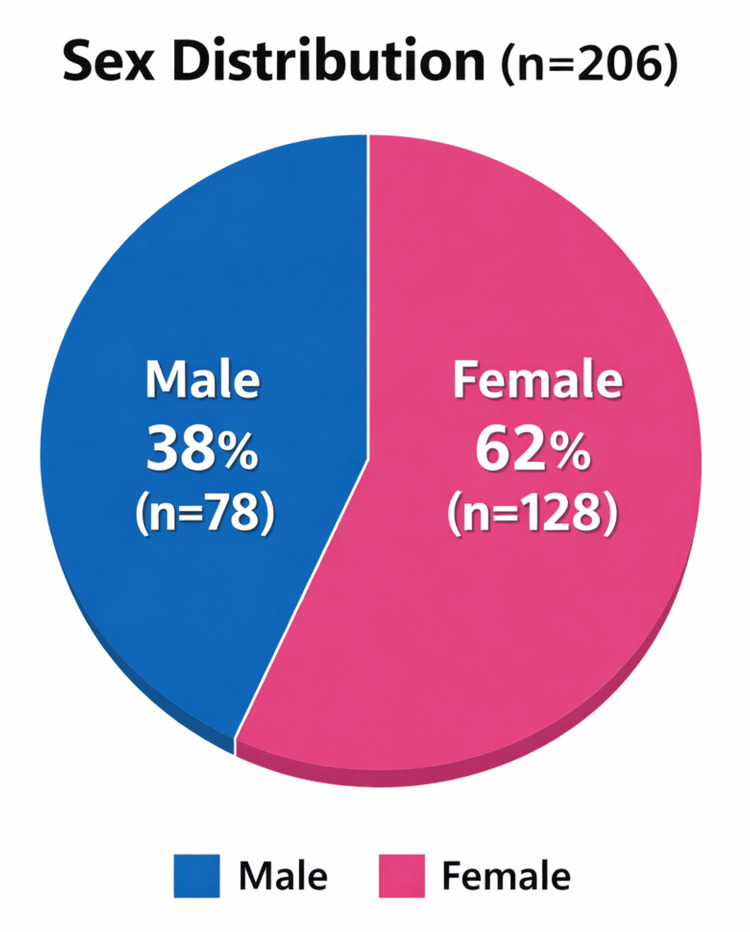
Sex distribution of healthcare professionals participating in the study The pie chart shows the sex distribution of healthcare professionals who participated in the study, indicating that 38% were male and 62% were female.

The sex distribution of the study participants is presented in Table 2 and Figure 3. A total of 206 healthcare professionals participated in the study, comprising individuals from various healthcare roles, including doctors, nurses, laboratory technicians, and HIOs. Among these participants, 78 (38%) were male, and 128 (62%) were female, demonstrating a higher representation of female healthcare professionals within the study population. This finding may reflect the overall gender composition in the healthcare workforce of the study setting, where females often constitute a significant proportion, particularly in nursing and allied health roles. The data illustrated in Figure 3 provides a visual representation of this distribution, highlighting the predominance of female participants compared to their male counterparts.

**Table 2:** Sex distribution of healthcare professionals participating in the study The table shows the sex distribution of healthcare professionals who participated in the study, indicating that 38% were male and 62% were female.

Sex	Number (n=206)	Percentage
Male	78	38%
Female	128	62%
Total	206	100%

Pre-test and post-test scores

The mean pre-test score was 13.33±2.64, which improved significantly to 17.43±0.69 post-test (p<0.0001). Nursing officers formed the largest group (27%), followed by PHCOs (22%) and doctors (20%). The intervention significantly enhanced awareness across all professional categories (Table 3).

**Table 3:** Pre-test and post-test scores of healthcare professionals according to cadre (n=206) The table represents the pre-test and post-test scores of 206 healthcare professionals, categorized according to their cadre. Data are presented as the number of participants (N), percentage of the total (%), and mean scores with standard deviation (mean±SD). The mean improvement indicates the difference between the post-test and pre-test scores. Statistical significance of the improvement was analyzed, with a p-value of <0.05 considered significant and <0.001 considered highly significant. PHCOs: primary healthcare officers; CHOs: community health officers; HIOs: health inspecting officers

Cadre/designation	N	%	Pre-test score (mean±SD)	Post-test score (mean±SD)	Mean improvement	P-value
Doctors	41	19.9	14.0±2.1	17.8±0.5	3.8	<0.001
Nursing officers	55	26.7	12.0±2.3	17.3±0.6	5.3	<0.001
Pharmacists	13	6.3	12.0±2.2	17.2±0.6	5.2	<0.001
Lab technicians	27	13.1	12.1±2.3	17.2±0.6	5.1	<0.001
PHCOs	46	22.3	13.0±2.0	17.5±0.5	4.5	<0.001
CHOs	8	3.9	13.2±1.5	17.4±0.5	4.2	<0.001
HIOs	16	7.8	12.5±2.1	17.3±0.5	4.8	<0.001
Total	206	100	13.0±2.3	17.4±0.6	4.4	<0.001

Table 4 presents the pre-test and post-test scores of healthcare professionals across different professional categories. A significant improvement in knowledge was observed in all groups following the intervention (p<0.0001), with the overall mean score increasing from 13.33±2.64 to 17.43±0.69.

**Table 4:** Pre-test and post-test scores of healthcare professionals following the intervention Data are presented as mean±standard deviation (mean±SD). Statistical analysis was performed using a paired t-test. A p-value of <0.05 was considered statistically significant.

Variable	Pre-test (mean±SD)	Post-test (mean±SD)	Paired t-test
Knowledge score	13.33±2.64	17.43±0.69	t=25.34; p<0.0001

The analysis of pre-test and post-test scores demonstrated a significant improvement in knowledge among all healthcare professionals following the educational intervention. The overall mean pre-test score was 13.33±2.64, which increased markedly to a post-test mean score of 17.43±0.69 (p<0.0001), reflecting the effectiveness of the intervention in enhancing awareness.

Among the different professional categories, nursing officers constituted the largest proportion of participants (27%), followed by PHCOs (22%) and doctors (20%). Improvement in scores was observed across all categories, indicating that the intervention was uniformly beneficial regardless of professional background. The largest absolute gains were noted among nursing officers and PHCOs, suggesting that structured educational programs can have a pronounced impact on frontline healthcare workers who frequently interact with patients.

Overall, these results underscore the effectiveness of targeted educational interventions in improving knowledge and awareness among healthcare professionals. The significant post-test improvements highlight the potential of such interventions to strengthen workforce competency, which may translate into better healthcare delivery and patient outcomes.

## Discussion

Breast and cervical cancers remain major contributors to cancer-related morbidity and mortality among women in India, posing a significant public health challenge, particularly in low-resource settings where early detection and timely treatment are often inadequate [1,2]. Strengthening the knowledge and capacity of healthcare professionals at the primary care level is crucial, as they represent the first point of contact for women seeking preventive and curative services [3].

The present study demonstrated that a structured educational intervention significantly improved knowledge of breast cancer, cervical cancer, and HPV vaccination among healthcare professionals working in PHCs of Davanagere district. At baseline, participants' understanding was limited, with a mean pre-test score of 13.33 out of 18, reflecting gaps in awareness of symptoms, risk factors, screening protocols, and HPV vaccination [4-6]. Following the educational session, the mean post-test score increased significantly to 17.43 (p<0.0001), confirming the effectiveness of structured training in rapidly enhancing knowledge [12,13].

The improvements in awareness of breast self-examination, Pap smear screening, and HPV vaccination are particularly noteworthy, as these are cornerstones of early detection and prevention. Enhanced knowledge among healthcare workers is likely to translate into improved community counselling and opportunistic screening [7-9]. Similar outcomes have been reported in prior studies conducted in Karnataka, Maharashtra, and other settings, supporting the universal applicability of targeted capacity-building programs [5,10-12].

The intervention benefited diverse professional cadres, including medical officers, nursing officers, PHCOs, pharmacists, laboratory technicians, CHOs, and HIOs, indicating that the module was well-designed, comprehensible, and relevant across all levels of PHC staff. Knowledgeable frontline staff can significantly influence early detection, preventive counselling, and timely referral, which is especially important given the critical role of PHCs in rural healthcare delivery [2,6,11].

Strengths of this study include its large and heterogeneous sample, representing 48 PHCs across the district, and the use of a pre-validated questionnaire in the local language (Kannada), which enhanced clarity, minimized misinterpretation, and improved reliability. Pre- and post-test assessment allowed for the objective quantification of knowledge gain, and the high participation rate increased representativeness [12,13].

Despite these strengths, the study has limitations. The single-group pre-post design lacked a control group, limiting causal inference as external factors may have influenced post-test results. Self-reported responses may be susceptible to social desirability bias, and knowledge improvements may not directly translate into behavioral changes or patient-level outcomes. Future studies should consider a controlled design and longitudinal follow-up to assess sustained impact on screening uptake and community health outcomes.

PHC professionals are strategically positioned to address barriers to early detection through counselling, opportunistic screening, and timely referrals. The results of this study suggest that structured educational interventions can empower PHC staff to take a proactive role in cancer prevention and early detection [1,9,12-14].

The findings of this study also support the broader objectives of national initiatives such as the National Program for Prevention and Control of Cancer, Diabetes, Cardiovascular Diseases, and Stroke, which emphasize capacity building of frontline healthcare workers. By improving knowledge and awareness, structured training interventions align with WHO strategies aimed at eliminating cervical cancer as a public health problem through increased HPV vaccination coverage and improved screening uptake [1,9,12-14].

In conclusion, the study demonstrates that structured, context-specific educational interventions can significantly enhance awareness of breast and cervical cancer and HPV vaccination among diverse PHC professionals. Such interventions are critical for improving early detection, preventive practices, and overall women's health outcomes in resource-limited settings.

Limitations of the study

Despite significant findings, this study has certain limitations. The quasi-experimental pre-test and post-test design lacked a control group, limiting the ability to attribute the observed improvements exclusively to the educational intervention. Self-administered questionnaires may have introduced response bias, as participants could overestimate their knowledge post-intervention.

The study assessed short-term knowledge gain immediately after the intervention; long-term retention of knowledge and actual changes in clinical practice or screening behavior were not evaluated. Additionally, the study was conducted only in PHCs of a single district, limiting generalizability to other healthcare settings or regions.

Finally, although the questionnaire was validated, it primarily measured awareness and knowledge rather than practical skills or competency in screening procedures, such as performing breast self-examination or counseling for HPV vaccination.

## Conclusions

The educational intervention effectively improved awareness of breast and cervical cancers and HPV vaccination among PHC-level healthcare workers. Regular, structured training sessions are essential to strengthen early cancer detection, opportunistic screening, and timely referral to higher centers, ultimately contributing to improved women's health outcomes and aligning with national and WHO cancer prevention strategies.
